# Serotonin Reuptake Inhibitor and Fluvoxamine-Induced Severe Hyponatremia in a 49-Year-Old Man

**DOI:** 10.1155/2009/585193

**Published:** 2009-10-13

**Authors:** Adel Gabriel

**Affiliations:** Airport Business Center, University of Calgary, 2000 Pegasus Road NE, Calgary, AB, Canada T2E 8K7

## Abstract

*Objectives*. To describe a case of fluvoxamine-induced severe hyponatremia, most likely due to abnormal antidiuretic hormone excretion (SIADH), and to discuss the implication for maintenance treatments for these patients. *Clinical Observations*. Although this syndrome had its incidence most commonly among the elderly, we report a case of severe hyponatremia (serum sodium <114 mmol/L), in a relatively young male. *Treatment*. Symptoms responded well to IV hyperosmolar sodium and to the discontinuation of fluvoxamine. This patient was maintained for treatment on an alternative Selective Serotonin Reuptake Inhibitor (SSRI), Citalopram, without developing recurrence of symptoms. *Outcome and Conclusion*. Protocols to monitor the maintenance treatments in high-risk patients may be needed to prevent recurrence of serious complications.

## 1. Introduction

SSRIs have gained widespread use in elderly depressed patients because of their favorable adverse effect profile. However numerous spontaneous reports of the (SIADH) have followed the increased use of selective serotonin reuptake inhibitors (SSRIs), especially among the elderly population. In a review by Liu et al., 1996, it was explained that although the published cases are very few, there were over 703 reported cases to authorities, to agencies, and to pharmaceutical companies. The majority of cases were reported with the use of flouxitine in over 75% of cases, and less commonly with other SSRIs, and most (83%) of the published cases involved patients with 65 years of age or more, as compared with 74% of the unpublished cases [1–3].

From reviewing literature, there are only three published cases, which were caused by fluvoxamine. In these cases, hyponatremia was severe (Na = 103–112 mmol/L), patients were treated with hypertonic saline infusion, and (SIADH) deemed as the most likely implicated causative mechanism [4]. The diagnosis of SIADH in these patients was made based on hyponatremia, low serum sodium, and high urine osmolalities. We report here a case of severe hyponatremia, which was associated with SSRI fluvoxamine administration in a relatively young male.

## 2. Case Report

A 49-year-old male admitted with severely depressed mood. He was communicating feelings of despair, guilty thoughts, and suicidal ideas. His symptoms started six months prior to this presentation after the death of his girl friend. There were significant vegetative symptoms, poor concentration, and severe anxiety for six months. In his psychiatric history, he was treated for depression and anxiety, for 3 years previously, with different antidepressants including the tricyclic, clomipramine 75 mg a day, without any untoward side effects, and he has been sober from heavy drinking for more than 10 years. In his family history, his mother suffered from depression and committed suicide when he was five years old. Patient has no history of cardiac, renal, endocrine, or liver disease. The primary diagnosis of major depression was confirmed by the MINI screen international neuropsychiatric interview schedule [5]. Following this hospital admission, clomipramine however was replaced with fluvoxamine. Fluvoxamine was titrated, with a starting dose of 50 mg, which was increased gradually to 150 mg a day. Ten days after starting fluvoxamine, he developed headaches, a grand mal seizure, and became lethargic. Lab work revealed severe hyponatremia (serum sodium 114 mmol/L), serum potassium 3.7 mmol/L, and renal functions were normal. Serum osmolality was 241 mmol/Kg and urine osmolality was 427 mmol/Kg, urine sodium 45 mmol/L, and urine potassium 7 mmol/L. On admission they were within normal, complete blood count, liver function tests, electrocardiography, elecrtoencephalography, and computed tomography of the head. Fluvoxamine was discontinued, and patient was placed on free water restriction, and hyperosmolar sodium infusion. Serum sodium returned to 139–141 mmol/L over the following four days, and patient's mental status improved gradually. Fluvoxamine was replaced by citalopram, which was increased gradually to 60 mg/day. Serum sodium remained stable within normal range for the 8 months following hospital discharge. During this period, sodium was monitored once on a daily basis for one week, then once weekly for another month and fortnightly for six months. No recurrence of hyponatremia was reported in this case.

## 3. Literature Review

Christensen et al. (1996) described three cases of hyponatraemia/syndrome of inappropriate secretion of antidiuretic hormone associated with Fluoxetine, paroxetine, and Citalopram, in three elderly women which improved with the discontinuation of the drugs and fluid restriction [6]. Masood et al. described a patient with hyponatremia associated with venlafaxine therapy [7], and Odeh et al. (2001) described an elderly woman who presented with severe symptomatic hyponatremia caused by the SIADH during therapy with Citalopram [8]. In these reports, a high incidence was consistently reported among the elderly 12.5% [9]. Hyponatremia and the syndrome of inappropriate antidiuretic hormone secretion (SIADH) have been also associated with several psychotropic drugs, for example, carbamazepine, neuroleptics, and tricyclic antidepressants. Although many case reports describe this association, few report the effect of rechallenge with another SSRI. There was a case in the literature for an elderly patient who developed hyponatremia with sertraline and SIADH when rechallenged with fluoxetine [10], and a case of an 82-year-old patient who was hospitalised for convulsions ten days after initiation of fluoxetine therapy for depression. She had severe hyponatremia and increased urine osmolarity suggesting SIADH [11]. Hyponatremia developed in (12%) of patients after 10 days approximately after initiation of paroxetine treatment. In this recent longitudinal study on ambulatory psychogeriatric patients (*n* = 75, and mean age 75.3), who received a diagnosis of major depressive episode, plasma sodium levels were monitored before initiating paroxetine therapy and after 1, 2, 4, 6, and 12 weeks of treatment. Lower body mass index and lower baseline plasma sodium level (<138 mmol/L) were significant risk factors for the development of hyponatremia [12].

A delayed recurrent hyponatremia, in an elderly patient, following the replacement of fluvoxamine with paroxetine was also reported, and authors suggested that plasma sodium concentrations must be monitored, not only in the first weeks of treatment, but throughout the full course [13].

## 4. Discussion

While SSRIs have been reported to cause SIADH, the actual incidence remains unclear. From the published case reports, the elderly may appear to be at higher risk of developing SIADH. According to symptomatic and laboratory presentation in this patient including lethargy, hyponatremia, the elevated urinary sodium, the inappropriately hyperosmolar urine, and the absence of cardiac, renal, endocrine, or liver disease, SIADH is suspected as contributing cause. Of the published reports, only three convincingly demonstrated a causative role of SSRI-induced SIADH by rechallenge. As a result, the published case reports cannot definitely establish a causal relationship. Physicians must recognize and ensure the proper monitoring parameters, namely, daily fluid intake, patient weight, and the serum sodium concentrations [14]. Age-related susceptibility to hyponatraemia may be explained by physiological changes in renal and endocrine function. The high prevalence of polymedication and pluripathology in the elderly was suggested by some of the contributing factors. For example, Rosner (2004) reported two cases of severe hyponatremia associated with combined use of thiazide diuretics and selective serotonin reuptake inhibitors [15]. Before being switched to fluvoxamine, our patient was on clomipramine, which is a well-known tricyclic antidepressant with potent SSRI properties. This may have predisposed him to be more vulnerable to the hyponatremic effect despite being younger in age group than those who were reported to suffer most.

Protocols for monitored maintenance treatments in high-risk patients need to be formalized in order to prevent recurrence and serious renal and neurological complications. However, an important question remains to be answered: should maintenance treatments monitoring become a formal process for these patients, how long monitoring should continue? This is very important to answer because of the fact that patients who suffer from depression may be switched from one antidepressant to another for different reasons, including intolerance to side effects, or poor efficacy, and may need prolonged periods of maintenance of the SSRIs therapy.

##  Disclosure

There was no funding from any pharmaceutical company to support writing this case report. Patient gave an informed consent to author for this publication.
